# Women and Public Health

**Published:** 1920-10-02

**Authors:** 


### Women and Public Health.

Very little space has been given in the English Press to the meeting of the International Council of Women which has just been concluded at Christiania. The gathering was a memorable one. Twenty-eight countries, says one of the British representatives who was present, sent delegates, and as a rule up to the number of ten for each country, the most notable and regrettable absentees being those from Germany. The German President wrote to explain that as long as Germany was not included in the League of Nations she would be meeting her fellow members in such a conference upon an inferior footing; a view not generally shared by the delegates from other countries, also outside the League.

But the important features of the gathering were the wave of earnestness which inspired all the discussions, the high ideals which moved the speakers, and the impressions conveyed to competent observers that the time is rapidly coming when the women of all countries will play a vital part in shaping opinion and framing laws on questions of public health, and, indeed, in all matters of social reform.

A refreshing difference of opinion was manifested in the consideration of the agenda, but the subjects were disposed of in a businesslike manner and with a real grasp of affairs. The question of the endowment of motherhood provoked a spirited debate, and in the end a resolution was passed supporting the principle of the endowment of necessitous motherhood. Another resolution concerned the suppression of the "traffic in women throughout the world," and urged that mandates by the League of Nations should be conditional on the abolition of State regulation of prostitutes within the mandatory territory—a drastic proposal which was unanimously adopted. Other resolutions urged the international interchange of public school teachers, and the right of women to choose their own nationality in marriage with an alien.

